# The use of focus groups to develop a culturally relevant quality of life tool for lymphatic filariasis in Bangladesh

**DOI:** 10.1007/s11136-013-0455-0

**Published:** 2013-06-22

**Authors:** Lynne Zeldenryk, Marion Gray, Susan Gordon, Rick Speare, Moazzem Hossain

**Affiliations:** 1 Occupational Therapy, School of Public Health, Tropical Medicine and Rehabilitation Sciences, James Cook University, Douglas, QLD 4811 Australia; 2 Occupational Therapy, University of the Sunshine Coast, Sippy Downs, QLD Australia; 3 Institute of Allergy and Clinical Immunology of Bangladesh, Dhaka, Bangladesh

**Keywords:** Cross-cultural, Instrument design, Focus groups, Research assistants, Lymphatic filariasis, Quality of life

## Abstract

**Purpose:**

The purpose of this study was to conduct focus groups to operationalise the construct of quality of life (QOL) for people living with lymphatic filariasis (LF) in Bangladesh to develop culturally valid items for a Bangladeshi LF QOL tool.

**Methods:**

Ten focus groups were conducted with a stratified purposeful sample (*n* = 60) of LF patients (3 focus groups, *n* = 17), doctors (1 focus group, *n* = 5), nurses (1 focus group, n = 6) and other hospital staff (1 focus group, *n* = 5), community leaders (2 focus groups, *n* = 14), community volunteer health workers (1 focus group, *n* = 5) and Bangladeshi LF researchers and planners (1 focus group, *n* = 8). Focus group methodology was informed by local culture in consultation with cultural mentors and local advisors, often going against standard focus group procedures. Data were collected through note taking, audio taping, transcripts, observational notes and a reflection diary. Open coding of transcript data was completed until data saturation was achieved.

**Results:**

Forty-three constructs were identified through the focus groups that had not previously been identified in the literature, including constructs relating to environmental supports and barriers, activities, participation and psychological impacts. There were marked differences between the impacts reported by different groups, highlighting the need for a comprehensive purposive sample. In particular, contributions from participants who would not traditionally be viewed as “experts” were vital.

**Conclusions:**

The use of focus groups strongly contributed to the operationalisation of the concept of QOL in Bangladesh for people living with LF. Use of literature review or expert opinion alone would have missed vital constructs.

## Introduction

### Lymphatic filariasis and the need for a quality of life measurement tool

Lymphatic filariasis (LF) is a neglected tropical disease and the leading cause of physical disability in the world, with 40 million people chronically disabled by the disease [1, 2]. It is spread via a number of different mosquito hosts, which vary depending on geographical location. The most common chronic clinical manifestations of the disease are lymphoedema of the limbs, scrotal hydrocele, acute filarial lymphangitis (AFL) and acute dermatolymphangioadenitis (ADLA). Acute filarial lymphangitis is an acute attack caused by the death of an adult worm that can cause mild fever, headaches and reversible distal lymphoedema. Acute dermatolymphangioadenitis is secondary bacterial infections which can cause cellulitis-like symptoms such as pain, fever and swelling and is more common than AFL [3]. Despite this, little is known about the disease’s impact on quality of life for people living with LF.

These clinical symptoms create a significant physical burden for people living with LF. The physical symptoms of the disease can be severely debilitating and impact on work, family, social and self-care activities [4]. External stigma relating to the disease creates significant psychological impacts, often leading to disengagement from important social and family roles [5]. As the disease is not fatal, there is decreased awareness of the importance, breadth and impact of LF disability for communities [6].

There remains an ongoing need to measure the impact of LF disability [7]. The impact of LF on the quality of life (QOL) of people living in LF endemic regions remains unknown. Tools currently being used in LF disability measurement include the ICF, WHOQOL-100, WHOQOL-Bref, WHODAS II, 5D7L and DLQI. These have been demonstrated to poorly measure common impacts of LF disability and to be culturally and linguistically inappropriate for use in rural Bangladesh [8, 9].

The purpose of a larger study by the authors of this paper was to develop a culturally appropriate QOL instrument for people living with LF disability in Bangladesh. One of the difficulties in developing a QOL tool is that evidence of the impact of LF on the QOL of people living with the disease remains scarce [5]. There is no evidence pertaining to Bangladesh. Whilst common impacts of the disease across a number of LF endemic regions have been reported [5], it was believed that greater operationalisation of the construct of QOL for people living with LF in Bangladesh was required.

This article describes the process by which focus groups (a) contributed to a greater understanding of the impact of LF on QOL in Bangladesh (b) informed the development of items for a Bangladesh-specific LF QOL measurement tool and (c) strengthened the clinical and cultural relevance of the tool. Finally, this paper compares the results of this study with item generation methods used in other studies.

### The use of focus groups for item generation and instrument development

Focus groups are a popular method to explore the experience of health-related QOL from the perspective of a community and target population. Focus groups aid in the conceptualisation and operationalisation of important constructs for QOL tools, informing the identification of themes and potential items within tools [10]. They are particularly useful for exploration of phenomena and experiences that are poorly understood, when the literature or expert opinion may not capture all relevant issues pertinent to the instruments intended target population [11–14].

In instrument development, a range of methods are used to identify, inform and develop questionnaire items [15, 16]. Traditionally, researchers have used literature reviews and consultation with “expert panels” to develop QOL tools through domain identification, item generation and instrument formation. In health research, “experts” are often defined as doctors and researchers working with people with a particular disease. Surveys where items have been generated through literature reviews and “expert panels” alone often fail to capture key issues of the phenomenon for the target population, as important key constructs are often missing from current evidence within the literature [12].

Focus groups provide the opportunity to explore constructs through the worldviews of the communities in which the tool will be used [10, 17]. Focus groups are a method where researchers can gain an “insider perspective” from those whom the instrument is intended to be used [18, 19]. Therefore, data collected from focus groups increase cultural and construct validity during tool development [13, 20].

## Methods

### Study design

This study is based on a sequential, mixed methods design for instrument development [21, 22] where separate stages of data collection and analysis are completed to generate items [23]. For this study, the first stage involved a literature review to identify key constructs central to the experience of QOL and LF globally. The methods and themes identified from the first phase have been published elsewhere [5, 24]. The second stage of the research process, the use of focus groups to operationalise QOL constructs for LF patients and generate items, is the focus of this article.

### Research context

This research study was conducted in the rural town of Saidpur, located in the Nilphamari region of northern Bangladesh. The Nilphamari region has a population of 1.5 million people and an average literacy of 25 % [25]. The region’s main industries are agriculture and farming. Nilphamari remains one of the poorest regions in Bangladesh [5]. Saidpur consists of 15 wards, each with a community leader. The majority of the population is Muslim (92.26 %) and Hindu (7.54 %) [26]. The filariasis hospital in Saidpur is the only filariasis hospital in the country and so patients travel from across the country to access treatment.

### Community consultation

As the researchers were foreign to Bangladesh, it was important to have local research assistants who could assist the researchers to build relationships locally and collaborate closely with the community [27]. The first author and six bi-lingual Bangladeshi research assistants lived in Saidpur for 1 month prior to commencing data collection to meet locals and observe local culture and lifestyles. This preliminary observation allowed for contextualisation of the research to occur [28] and informed the specific nuances of the focus group methods for this study. Advice was sought from local LF experts who are actively working in the field and also from a number of doctors outside of the LF field who could comment on the health system and structure in Bangladesh.

### Sampling

Stratified purposeful sampling was used to ensure key informants were sourced who could provide informed insight. Local advisors (key leaders in the LF field in Bangladesh and local health staff who had an awareness of both the institutional and community health-care systems) indicated five target populations that could provide good understanding of the impact of LF within a Bangladeshi context: (1) people living with LF disability in the community, (2) doctors, nurses and other health staff directly involved in delivering health care to LF patients, (3) community leaders, (4) community volunteer health workers, (5) Bangladeshi LF researchers and planners. The involvement of participants from the regions where the final tool is to be used increased the cultural relevance of the final tool [29].

In total, 10 focus groups were conducted with 60 participants. Size of focus groups ranged between five and eight participants per group. Groups were as homogenous as possible (structured by professional or community role with patients grouped by gender), to minimise the impact of social hierarchies that could inhibit discussion [29]. The demographics of the focus groups were as follows: two female patient groups (*n* = 11), one male patient focus group (*n* = 6), one community volunteer health worker group (*n* = 5), two community leaders groups (*n* = 14), one group of doctors (*n* = 5), one group of nurses (*n* = 6), one group of other health staff from the Filaria Hospital (project officers, administrators and laboratory technicians) (*n* = 5) and one group of LF researchers/health programmers (*n* = 8). For the patient focus groups, moderators were gender matched to ensure open discussion regarding gender sensitive topics (such as impacts on relationships). See Tables 1 and 2 for participant demographics.

**Table 1:** Focus group demographics—health professionals, researchers and community workers

Group (*n*)	Gender (*n*)	Years working with people with LF
Doctors (5)	M (4) F (1)	>1 (1) 1–4 (0) 5–10 (1) 11–15 (3)
Nurses (6)	M (0) F (6)	Unrecorded (6)
Other hospital staff (5)	M (4) F (1)	>1 (0) 1–4 (3) 5–10 (0) 11–15 (1) 16+ (1)
Community volunteer health workers (5)	M (2) F (3)	>1 (0) 1–4 (1) 5–10 (1) 11–15 (0) 16+ (2) Unrecorded (1)
Community leaders (14)	M (11) F (3)	Unrecorded (14)
LF researchers/programmers (8)	M (7) F (1)	Unrecorded (8)

**Table 2:** Focus group demographics—patient groups

Group (*n*)	Age	Disease stage/presentation	Location of residence
Male (6)	20–29 (2) 30–39 (1) 40–49 (1) 50–59 (1) 60–69 (0) 70+ (1)	Lymphoedema only stage unknown (3) Lymphoedema and hydrocele (2) Hydrocele only (1)	Village (3) Town (3)
Female (11)	20–29 (0), 30–39 (2), 40–49 (4), 50–59 (5)	Stage 1 lymphoedema (1) Stage 2 lymphoedema (4) Stage 3 lymphoedema (3) Stage 4 lymphoedema (1) Stage 5 lymphoedema (1) Bilateral lymphoedema stage 2 & 3 (1)	Village (10) Town (1)

All participants provided informed consent to participate in this study, through information and consent forms. Information and consent forms were read to those who were illiterate who then provided a mark of consent if they could not sign their name. Participants were informed that they were allowed to remove themselves from the study at any stage. Human ethics approval was gained from James Cook University Human Ethics committee (reference: H3710) and the Bangladesh Medical Research Council (reference: BMRC/NREC/2010-2013/914).

### Focus group design

Culturally specific focus group methodology was developed for this project based on local advice and observation of local communication styles and hosting methods. This included the serving of food and “cha” (tea) by the principle investigator at the beginning of the focus group, followed by “gossip” or general talk about people’s families and backgrounds. This method followed the local custom of hosting, where hosts serve food and drink (which they do not participate in the consumption of) before gossiping with guests. These methods reinforced the researcher’s with respect to the guests (focus group participants) and allowed for rapport to be built prior to the formality of the focus group questions. Level of formality in focus groups differed depending on the group. For example, the LF expert group and the doctors’ group were run very formally and with English consent forms, as we had been advised that this indicated respect for their status and educational level. In comparison, focus groups with nurses and patients were less formal and more aligned with “social gossiping”, a style of discussion participants had more experience of and felt more comfortable with.

Following refreshments, focus groups were conducted using semi-structured questions to prompt discussion of the impacts of LF (see Table 3). As the group discussion occurred, a list of impacts were recorded on the board under a number of categories, each listed on a separate sheet of paper stuck to the wall of the room: activities, participation, environmental (stigma/culture/systems) barriers, psychological, medical and other impacts. These categories were pre-identified from the previous literature review into LF disability. This process allowed participants to member check the moderator’s interpretation of their discussions as the themes arose, important to achieving participant endorsement of the researchers’ interpretation of the data [28]. This method was used in all groups except the patients as many were illiterate and could not participate in the member checking exercise.

**Table 3:** Focus group questions

In what ways do you think LF affects you/the lives of people that you work with?
How does LF affect your/people’s ability to complete daily activities?
Home, work, school
How does LF affect your/people’s ability participate
Family roles, community activities
How well are you/LF patients supported
By their families, community, health services
How does having LF impact on your/the patients relationship with
Families (*prompt: spouse, in laws, children)*
Friends, ability to get married
How do people perceive and treat/interact with you/LF patients?
Within the patients family, community, health system
In what ways does LF affect your/the patient’s psychological health? In what ways?
Are there any aspects of the LF that you/your patients find difficult to accept? What are the most difficult things to accept?
Finally, in what other ways do you think that LF changes your life/the lives of people living with the condition?

### Focus group moderation

Four research assistants assisted with focus group moderation, with three more assisting with research organisation and cultural advice. Two of the moderators had completed master’s degrees in social science and had experience in focus group methodologies. These two moderators conducted the more formal focus groups with the LF experts, doctors, nurses, health workers, community leaders and community health workers. Two other research assistants conducted the patient focus groups. These moderators had less experience in focus group methods, however, had undergraduate degrees in health and disability and hence, could relate more easily with the focus group participants for the patient groups. Regardless of their level of skill or experience, all moderators received extensive training in the purpose of the study and research ethics and conducted the focus groups using the same moderator guides. The use of academic papers as additional information to train moderators was initially trialled; however, this caused confusion often, as many methods were not relevant in the Saidpur context. The development of a moderator guide for this project, with local considerations informing methods, clarified moderation style and ensured consistency of purpose and focus group topics across groups. Moderators worked in close consultation with the first author and community advisors to explore and identify the interviewing skills that were culturally appropriate to the different participant groups and to refine the final version of the focus group plan. Moderators completed role-plays and practised focus group methods with the first author and as a group, to practise phrasing, time management, prompts/probing methods and gender-specific research methods. This assisted in ensuring that moderation styles were similar regardless of the experience level of moderators.

### Data collection and analysis

Focus group discussions were audio taped, transcribed and translated by the focus group moderator immediately following the focus group. An independent bi-lingual research assistant conducted spot checking of transcript translations.

Following each focus group, the moderator, interpreter and first author collated observational notes and the notes of themes taken during focus groups. The first author discussed her observational notes to check for correct interpretation of concepts and cultural biases. Reflection notes were also taken by the first author to continually reflect on her role as a foreigner within the research environment, which could affect outcomes and influence responses and interpretation [30]. A decision trail was kept throughout these discussions, and notes were taken to track key observations and decisions regarding interpretations [31]. Data collection continued until saturation of data occurred [32].

Content analysis was performed through open coding. Open coding is where raw data are analysed through a detailed line-by-line review of the transcript to identify themes or phenomena of interest which are then ascribed codes [17]. Sections of text can then be compared and compiled where codes are recurrent or relate. In this study, the first author ascribed codes for sections of text from the transcripts, allowing themes to emerge from the data [28] before cross-checking interpretation with research assistants along the way to minimise misinterpretations [33]. Codes for common themes were generated allowing frequency of themes across transcripts to be collated. Peer review of the analysis occurred where two other researchers from the team independently analysed transcripts using open coding and then cross-checked for discrepancies. A decision trail was kept of coding decisions made through cross-checking of analysis with the team [34].

## Results

The use of focus groups to explore the impact of LF disability and QOL in Bangladesh resulted in the generation of 43 new themes that the literature review had not previously identified (See Table 4). New themes arose within the environmental barriers and support domain (*n* = 23), the activity and participation domain (*n* = 11), in other impacts (*n* = 8) and the psychological domain (*n* = 4). Interestingly, no new themes arose from patient focus groups, indicating data saturation had occurred.

**Table 4:** Mapping of themes that arose through literature review and focus groups

Domain	Themes from literature review	New themes from focus groups
Psychological	Depression	Feeling useless/valueless
	Feelings of shame/humiliation	Stress/anxiety
	Low self-esteem/inferiority	Feeling unloved
	Feeling unattractive/poor body image	Helpless
	Ability to cope/strategies	
	Grief/loss of former self	
	Fear	
	Wishing they were dead	
	Embarrassment	
	Feeling isolated	
	Hopelessness	
	Frustration	
	Feeling inadequate	
	Feeling like a burden	
Activities & Participation	Sexual functioning	Fine motor skills
	Work	Eating
	Mobility	Gather/farm food
	Childcare	Company/time spent with family
	Domestic chores	Company/time spent with others/friends
	Catch transport/cycling, etc.	Patient separates themselves from community
	Self-care	Patient separate’s themselves from family
	Sleep	Heavy work
	Marriageability	Shopping–community CADLs
	Personal relationships	Takes longer to complete daily activities
	Attend social events	Role changes
	Ability to go to school	
Environment	Teasing	LF reduced social status of family
	Avoided by others	Respect from community
	LF reduced social status	Respect from family
	Stigma within family	Fear of contagion from others
	Stigma within community	Acceptance within community
	Families as carers	Acceptance within family
	Treatment availability	Support in community
	Expense of treatment	Stigma/barrier in government system
	Location of treatment	Neglected by family
	Stigma within health system	Pitied by others
	Stigma within school system	Inappropriate/dangerous treatment
	Access to support groups	Abandoned by family
	Hygienic home conditions	Abandoned by community
	Hygienic work conditions	No place within society
		Separated from family
		Separated from community
		Fear/mistrust of health services
		Housebound
		Hated by others
		Relationships with family deteriorate
		LF bringing shame to family
		Others feel annoyed by them
		Criticised by others
Personal/other	Poverty	Abuse
	Education status	Smell of wounds
	Pain	Weakness
		Bedbound
		Itching
Total items	46	43 new items

Themes arose in different frequencies across the focus groups, with many occurring in a few groups (see Table 5). The nurses’ group frequently discussed the impact of LF on relationships, especially regarding impact of LF spousal relationships and gender issues. In contrast, LF researchers/program staff talked more about the impact of the disease at a systems level, e.g., stigma/barriers in government systems and inappropriate/dangerous treatment. Community leaders and female patient groups talked most about the impact of the disease on the patients’ ability to spend time with family, whilst community health workers focused more on the community’s fear of contagion. Abuse was not discussed broadly across the groups. However, the other health staff from the hospital, who work most closely with families and saw first hand how families treated patients, discussed abuse of patients most frequently. See Table 6 for supporting quotes for new themes.

**Table 5:** Mapping of new themes as they arose in separate focus groups

New theme	Drs^1^	Nurses^2^	Other^3^	R/P^4^	CL #1^5^	CL #2^6^	CH^7^	Total
*Psychological*								
Feeling useless/valueless	0	1	6	1	0	0	1	9
Stress/anxiety	0	0	0	0	2	0	3	5
Feeling unloved	0	0	1	0	0	0	1	2
Helpless	0	0	2	0	0	0	0	2
*Activities & participation*								
Fine motor skills	0	0	2	4	0	0	2	8
Eating	1	0	1	0	0	6	2	10
Gather/farm food	1	2	4	0	0	2	2	11
Company/time spent with family	5	5	8	11	14	16	3	62
Company/time spent with others/friends	0	0	1	2	0	4	1	8
Patient separates from community	5	2	1	11	2	3	1	25
Patient separates from family	1	1	1	3	1	3	2	12
Heavy work	1	0	0	0	1	0	2	4
Shopping	0	0	0	0	0	1	0	1
Takes longer to complete daily activities	0	1	2	0	0	0	0	3
Role changes	0	0	0	0	6	2	2	10
*Environment*								
LF reduced social status of family	0	4	1	2	11	1	0	19
Respect from community	1	3	11	0	8	5	3	31
Respect from family	0	5	6	1	5	6	1	24
Fear of contagion from others	3	7	8	8	4	2	11	43
Acceptance within community	2	0	6	0	6	0	0	14
Acceptance within family	1	1	6	0	0	0	0	8
Support in community	0	0	0	0	0	1	0	1
Stigma/barrier in government system	0	0	3	17	0	2	0	22
Neglected by family	2	5	2	2	1	14	2	28
Pitied by others	0	0	0	0	0	6	0	6
Inappropriate/dangerous treatment	1	5	0	15	2	2	4	29
Abandoned by family	1	5	4	0	3	1	1	15
Abandoned by community	1	0	6	0	4	2	1	14
No place within society	1	1	1	0	0	0	0	3
Separated from family	0	5	4	7	0	7	3	26
Separated from community	2	4	2	1	0	7	5	21
Fear/mistrust of health services	1	1	2	0	0	0	0	4
Housebound	0	0	0	0	0	0	1	1
Hated by others	0	0	0	1	0	15	0	16
Relationships with family deteriorate	3	9	1	4	3	7	8	35
LF bringing shame to family	0	2	0	0	0	0	0	2
Others feel annoyed by them	1	1	0	0	0	1	7	10
Criticised by others	0	0	2	0	0	0	2	4
*Other*								
Abuse	0	5	11	0	7	3	0	26
Smell of wounds	3	0	0	9	0	0	0	12
Weakness	0	0	0	0	1	3	2	6
Bedbound	0	0	1	0	0	0	2	3
Itching	1	0	0	0	0	0	2	3

1 = Doctors at Filaria Hospital; 2 = nurses at Filaria hospital; 3 = other staff at Filaria Hospital; 4 = LF researchers/planners; 5 = community leaders FG #1; 6 = community leaders FG #2; 7 = community health workers

**Table 6:** Supporting quotes relating to new themes

Domain	Theme	Supporting Quote
Psychological	Feeling useless/valueless	*A concept grows in patients mind that it’s true that I am really a useless person*
	Stress/anxiety	*Tension [stress}, patient becomes fretful*
	Feeling unloved	*They think, no one loves or accepts me*
	Helpless	*Patient becomes hopeless and they breakdown*
Activities and Participation	Fine motor skills	*You are not able to do any type of hand related work*
	Eating	*It has been seen that he is not able to eat by his/her own [self]*
	Gather/farm food	*They can’t do their own farming work*
	Company/time spent with family	*No* *one… wants to talk with the [LF] patient or accompany them/give them company*
	Company/time spent with others/friends	*Patient thinks…I cannot gossip and talk with people.*
	Patient separates from community	*If the swelling and smell is very bad then the patient isolates himself from the others.*
	Patient separates from family	*They want to live alone, separated life. They become detached from family*
	Heavy work	*They are not able to do any heavy work*
	Shopping–community CADLs	*Also he cannot go shopping*
	Takes longer to complete daily activities	*They take more time to do what we do normally*
	Role changes	*He [LF patient] lost the participation [in family] that should be his*
Environment	LF reduced social status of family	*If a girl is affected then the marriage will happen with… a poorer family*
	Respect from community	*In society… they [LF patient] become less important and less important*
	Respect from family	*“You are not able to work then what is the value of your opinion?”* – *family members think like that.*
	Fear of contagion from others	*[People in the community say] you can be affected by LF if you mix with the patient.*
	Acceptance within community	*They often lose their social and communal acceptance.*
	Acceptance within family	*They haven’t any participation [within family] because they haven’t the acceptance*
	Support in community	*Sometimes the community helps the patient, try and help the patient*
	Stigma/barrier in government system	*They are not able to work [in the government sector]. They are not allowed abroad…it is a problem.*
	Neglected by family	*I saw that no one took care of him. He was laying in the corner of the house with a blanket.*
	Pitied by others	*Is very difficult to accept that people pity them. People don’t want for other people to pity them.*
	Inappropriate/dangerous treatment	*Doctors do not treat the LF patient [properly]… because they don’t know about LF, how to treat it.*
	Abandoned by family	*I saw that patient become outcaste/separated from his family*
	Abandoned by community	*They are separated from community and helpless.*
	No place within society	*He has no place in society*
	Separated from family	*A patient becomes separated from family.*
	Separated from community	*Family say “don’t go out, stay in your room”.*
	Fear/mistrust of health services	*They [healers] give them [LF Patient] a frightful concept about LF and the hospital*
	Housebound	*The patient feels shy to come out from the house.*
	Hated by others	*Generally all people hate an LF patient*
	Relationships with family deteriorate	*Of course the relationships become worse between patient and family*
	LF bringing shame to family	*Family think that it is a shame to show/expose him [LF patient] to the relatives or outside*
	Others feel annoyed by them	*People support the patient for 1 or 2 times but after that people become annoyed by them*
	Criticised by others	*Family criticise the patient*
Other	Abuse	*Family members…abuse*
	Smell of wounds	*When the patient suffering at stages 4*-*5 then it smells bad*
	Weakness	*They feel very weak*
	Bedbound	*He/she lay down in bed all the day*
	Itching	*Itching was a problem*

There were marked differences between the female and male patient focus groups. The women reported a much greater impact of LF on their daily lives than the men. Women attributed the impacts to many issues of stigma; they reported being hated by others (discussed 12 times in one focus group compared to only two times in the male group) and a reduction in their family’s social status (discussed 10 times in one female focus group but not at all within the male group).

## Limitations

Focus groups were conducted in the one region in Bangladesh that has a filarial hospital and so findings may be specific to the region. One focus group per responder type may have led to idiosyncrasies in themes, particularly for the one group of male patients. This was minimised by the use of triangulation in data collection using literature review and field testing through cognitive interviewing (conducted later in the research process).

Member checking with patient groups in the study was limited due to illiteracy. Member checking could not be done verbally after focus groups as most had travelled long distances and had limited time. Findings could have been different in patient groups with greater literacy levels (potentially less stigma); however, sampling reflects the population who are most at risk of LF- those who are poorer and with less education. Pre-identified categories on the boards may have led participants responses, however, pre-testing of broader focus group questions which did not specifically step through each of the categories proved ineffective, as the questioning was deemed “too broad” and vague for usual direct Bangladeshi communication styles.

Having different moderators of various experience levels could have resulted in inconsistent moderation styles. Inconsistency was minimised with the use of moderation guides and training. The use of less experienced moderators who had greater experience working with people with disabilities worked effectively as their experiences and knowledge in disability, and how it affects daily life for people in Bangladesh, strengthened the style of questioning and probing around these issues. Gender-matched moderators added to the comfort of the focus group participants in discussing more sensitive topics. Many participants knew and worked with each other, and there is the possibility that respondent social desirability bias could have affected responses to questions. This bias was minimised through moderators prompting discussions in the second person, allowing for “saving face” to occur [27].

Researcher bias from the first author could have influenced the data analysis and interpretation. When a researcher is entrenched in a certain research context, subjectivity is affected and bias cannot be wholly removed [30]. However, the use of the reflective diary, team analysis and the documentation of an audit trail throughout the research process assisted to minimise this bias.

The participant sample may not have represented all the experiences of people living with LF disability in Bangladesh, which is a limitation of using focus groups in instrument design [35]. However, the mixed methods approach used in this study allowed data collection to be completed until redundancy in data was achieved.

## Discussion

The identification of 43 new themes demonstrates the importance of community consultation and in-country data collection to operationalise key constructs and to strengthen validity of QOL instruments in culturally diverse contexts.

High-quality evidence of the impacts on QOL for people living with LF disability is scarce [5]. This study adds much to the understanding of the needs and experiences of people living with LF disability in Bangladesh. The use of literature review alone would have missed a number of important constructs relevant to LF disability and QOL, particularly in terms of impact of the disability on daily activities and participation and environmental barriers (such as government, community and family attitudes). This study provides greater evidence of the non-medical impacts of LF disability, particularly regarding environmental barriers such as local attitudes and the impacts of disability on daily life that LF programs in Bangladesh (and many other endemic regions) currently fail to address. Had the instruments’ items been informed solely by literature review, operationalisation of the concepts would have been insufficient and important key constructs would have been missed in the final tool. This information is vital for informing holistic interventions that address the social and psychological burden of disease. The engagement of local community leaders and community health workers who would not traditionally be viewed as “experts” greatly strengthened the validity of the tool for the local context. In this way, focus groups strengthen the construct validity and cultural relevance of instruments [27].

Carefully considered purposeful sampling was used to ensure participants could provide a full picture of the impacts from a number of perspectives [17]. Distinctive concepts arose in different groups and a breadth of discussion and viewpoints were shared. The contribution of nurses and community leaders, not traditionally regarded as “experts”, added much to conceptualisation of constructs. Whilst patient groups did not add any additional themes, their focus groups allowed for data saturation to occur and, importantly, confirmed the themes that arose from other groups were relevant for the experiences of people living with LF in Bangladesh. The patient groups included a spread of ages and stages/presentations of disease, allowing for perspectives from those with various experiences of the disease. It remains essential to include patients in the development of patient reported outcome measures to ensure that concepts included in the final tool are relevant to those living with the disease. In line with the philosophy of “nothing about us without us”, which advocates that interventions and planning must take into account the perspectives of those living with disability [36], it is argued that instrument development should also account for patient perspectives.

The findings of this study highlight the importance of methodological rigour in instrument design, particularly in cross-cultural contexts. Cross-cultural focus group methods require flexible and carefully considered approaches [20]. Culturally informed methods in this study were complex and multidimensional. Grouping participants in “like” groups removed social norms that could create barriers to open discussion. Going against conventional focus group methods, which advocates for participants to be strangers [37], many of our focus group participants knew each other. Similar to other studies [38], our participant sample was limited to those working in the field and so they often worked together. We found that participants had similar roles/experience/status and felt at ease, leading to an open and easily facilitated discussion. This finding reflects that of Strickland’s study [29] in Northwest Indian communities where speaking to a group who know each other was, culturally, the most appropriate method. It is important for focus group moderators to have a strong understanding of local context and the different communication styles and cultural influences that could impact on different participants’ involvement in the discussion [16, 39].

Moderation styles were altered in consideration of the social status of each of the participant groups. This method was particularly important in Bangladesh, where social hierarchies impact on who can speak when, how and in what terms in formal discussions. There remains minimal discussion in the literature around how to modify focus group questions and moderation styles to suit different cultural groups [29] or in this case, how to alter styles for different participant groups in the one culture. Our study greatly benefitted from having flexible moderation styles that could be altered for the audience in order to build rapport in the groups. In many ways, formal focus group methods are foreign to certain communities [27]. Our study’s use of carefully selected moderation styles, which reflected local norms of communication and “gossip”, relaxed participants who had previously had no experience with focus groups.

Likewise, focus groups with community leaders were held around a “community leaders’ consultation day” which included an official welcome, a shared formal meal, time for community leaders to meet as a group (something which was rare) and planned times and spaces for prayer during the day. The careful planning of this event in line with cultural norms helped build trusting relationships between the community and the researcher. Whilst time intensive, the establishment of rapport in culturally diverse groups remains vital to gaining good data [40]. Gaining trust and respectful relationships with participants are both a wise research strategy [39, 41] and an important ethical consideration in community-based research.

## Conclusion

This study demonstrates the importance of in-country data collection to the operationalisation of QOL concepts for instrument development in cross-cultural settings. Overall, the quality of the LF QOL tool was immensely improved by having focus groups supplement themes from the literature review. In particular, the use of purposeful sampling assisted in seeking a diverse range of perspectives on the impacts of LF disability on QOL in Bangladesh. The value of culturally informed research methods greatly strengthened local engagement with the project allowing for open discussion and greater insight into the impact of LF disability locally.
